# MAP-SNN: Mapping spike activities with multiplicity, adaptability, and plasticity into bio-plausible spiking neural networks

**DOI:** 10.3389/fnins.2022.945037

**Published:** 2022-09-20

**Authors:** Chengting Yu, Yangkai Du, Mufeng Chen, Aili Wang, Gaoang Wang, Erping Li

**Affiliations:** ^1^College of Information Science and Electronic Engineering, Zhejiang University, Hangzhou, China; ^2^Zhejiang University - University of Illinois at Urbana-Champaign Institute, Zhejiang University, Haining, China; ^3^College of Computer Science and Technology, Zhejiang University, Hangzhou, China

**Keywords:** spiking neural network (SNN), leaky integrate-and-fire (LIF) neuron, multiple spike pattern (MSP), spike frequency adaption (SFA), state-free synaptic response model (SFSRM), neuromorphic recognition, backpropagation (BP)

## Abstract

Spiking Neural Networks (SNNs) are considered more biologically realistic and power-efficient as they imitate the fundamental mechanism of the human brain. Backpropagation (BP) based SNN learning algorithms that utilize deep learning frameworks have achieved good performance. However, those BP-based algorithms partially ignore bio-interpretability. In modeling spike activity for biological plausible BP-based SNNs, we examine three properties: multiplicity, adaptability, and plasticity (MAP). Regarding multiplicity, we propose a Multiple-Spike Pattern (MSP) with multiple-spike transmission to improve model robustness in discrete time iterations. To realize adaptability, we adopt Spike Frequency Adaption (SFA) under MSP to reduce spike activities for enhanced efficiency. For plasticity, we propose a trainable state-free synapse that models spike response current to increase the diversity of spiking neurons for temporal feature extraction. The proposed SNN model achieves competitive performances on the N-MNIST and SHD neuromorphic datasets. In addition, experimental results demonstrate that the proposed three aspects are significant to iterative robustness, spike efficiency, and the capacity to extract spikes' temporal features. In summary, this study presents a realistic approach for bio-inspired spike activity with MAP, presenting a novel neuromorphic perspective for incorporating biological properties into spiking neural networks.

## 1. Introduction

Spiking Neural Networks (SNNs) are presented as noise-robust third-generation neural networks due to their biological plausibility (Maass, 1997). Similar to the brain's communication mechanism, the SNNs send discrete action potentials (spikes) across adaptive synapses to interpret information. Therefore, it is hoped that the study of SNNs will assist in revealing the functioning mechanism of the mind and intellect (Ghosh-Dastidar and Adeli, 2009). Moreover, the event-driven nature of SNNs makes them potentially energy-efficient on neuromorphic hardware and sensors (Liu and Delbruck, 2010; Vanarse et al., 2016).

However, designing and analyzing SNN training algorithms is difficult. The asynchronous and discontinuous nature of SNNs makes it difficult to use the well-established backpropagation (BP) approach for practical training (Pfeiffer and Pfeil, 2018). Recent studies have developed a pseudo-derivative approach to overcome the non-differentiable issue, enabling SNN to be directly trained using BP (Lee et al., 2016; Wu et al., 2018; Neftci et al., 2019). It deserves to mention that Wu et al. (2018) presented the Spatio-Temporal Backpropagation (STBP) technique, which for the first time translated the discretized Leaky-Integrate-and-Fire (LIF) neural model into the Pytorch framework (Paszke et al., 2019), significantly advancing the advancement of direct training approaches. Since then, several research works on SNN algorithms based on the Backpropagation-Through-Time (BPTT) algorithms and the LIF neural model have appeared, consistently showing the capability and performance of SNN (Lee et al., 2016; Shrestha and Orchard, 2018; Wu et al., 2018, 2019; Neftci et al., 2019; Yin et al., 2020; Fang et al., 2021). We refer to these direct training techniques as BP-based SNN, or BP-SNN for short. These BP-based SNNs implement the fundamental notion of Recurrent Neural Networks (RNNs) by transforming spiking neurons into an iterative model and replicating neural activity with discrete time iterations. With BP-based learning methods, SNN models can be applied on a larger scale inside existing deep learning frameworks for improved performance (Wu et al., 2019; Woźniak et al., 2020). However, we observed that the widely used discrete LIF model differs significantly from the definition in its differential form, and that the dynamics of neuron membrane potential and spike activity pose a critical discretization issue. This paper explores the intrinsic limitations of two broadly used discrete LIF models, the soft-reset model (Han et al., 2020) and the hard-reset model (Wu et al., 2018), and redefines the applicable conditions and underlying assumptions for their implementation. Mathematically, we re-derived the numerical solution process of the LIF neuron model to obtain an accurate discretization model (which we refer to as the standard numerical model), and by adding constraints, the standard discrete model is simplified to obtain approximate models under various assumptions and their potential errors.

**Figure 1:**
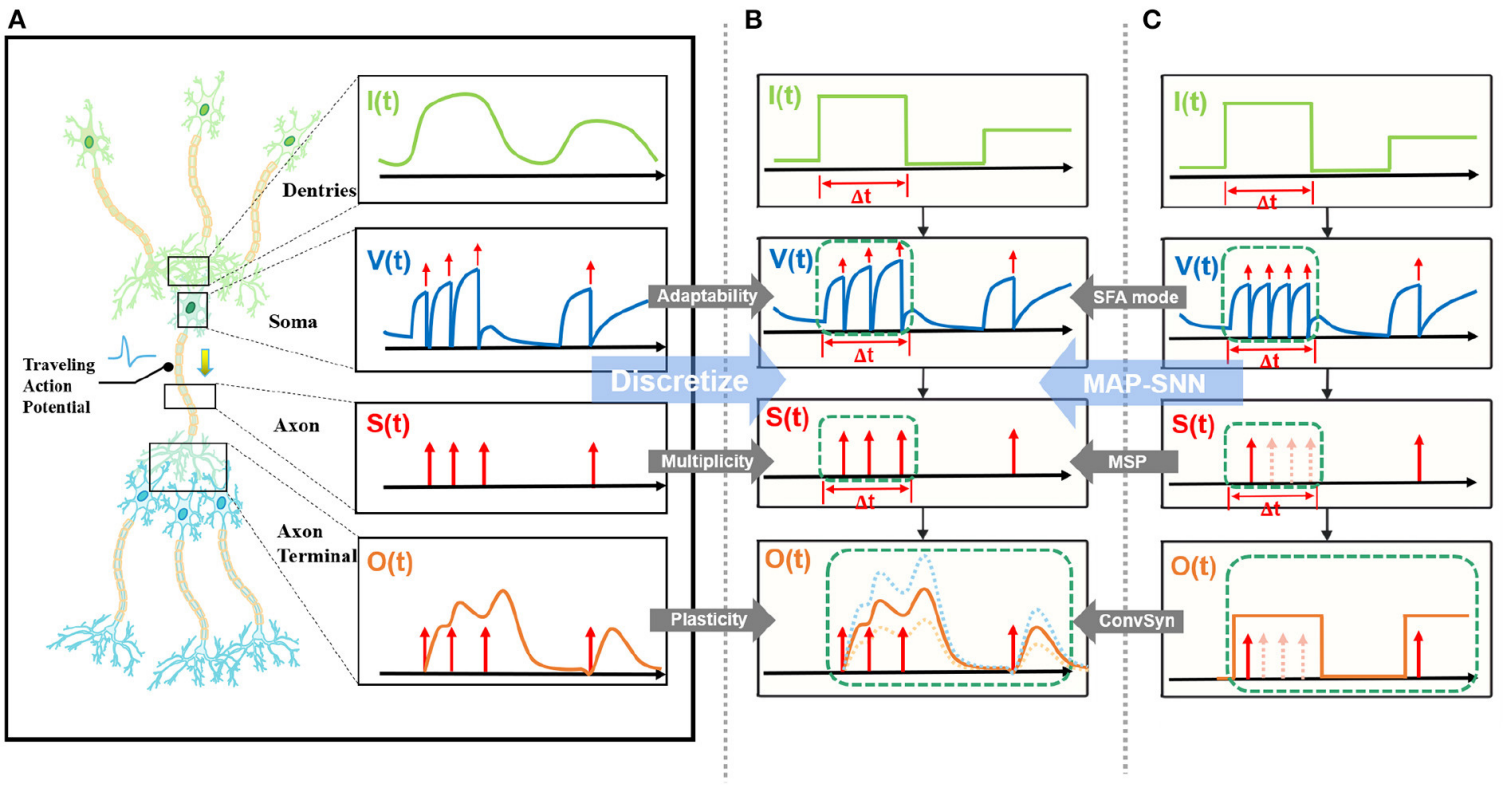
MAP-SNN overview. **(A)** Modeling neural model mathematically with Dendrites, Soma and Axon. The four variables *I*, *V*, *S*, and *O* describe the input current, the membrane potential, the spike transmission, and the output current concerning time, respectively. **(B)** MAP-SNN: Discretized spike activities with MAP properties. **(C)** Discretized spike activities without proposed improvements.

State-of-the-art bio-inspired SNNs reveal the potential of biological characteristics with better performance, such as the delayed Spike Response Model (SRM) for synaptic expressions (Shrestha and Orchard, 2018), biologically plausible BPTT for reward-based online learning (Bellec et al., 2020), Lateral Interactions for intra-layer connections (Cheng et al., 2020), and Nematode Neural Circuits Policies for auditable autonomy (Lechner et al., 2020), guiding researchers to delve into more biological realities. Focusing on the biological features of spiking activity, Multiplicity, Adaptability, and Plasticity, we propose three modules and integrate them to accomplish reliable and stable neural data classification tasks with varying temporal moderation, as shown in Figure 1. Specifically, the multiplicity prompts us to evaluate the prospect of multiple spikes happening within a short period of time, enabling us to consider the constraints of SNNs based on binary spike transmission. Initially, BPTT-based SNNs needed a high number of time steps to verify that discrete models and LIF neuron dynamics corresponded (Wu et al., 2018). However, recent work has reduced the number of time steps to make inference computation faster and more efficient, while also lowering SNN calculation latency (Wu et al., 2019; Yin et al., 2020; Chowdhury et al., 2021; Fang et al., 2021). When total time is constant, a lesser number of time steps corresponds to a larger step length (Δ*t*), which may violate the pre-restriction (Δ*t* → 0) of the discrete LIF model. In accordance with the original aim of neuron dynamics, we argue that SNNs using binary signals should be scalable to integers. Xu et al. (2020) initiated multiple spike codes at the hardware level and offered time compression to shorten the computational latency of inference on pre-trained models. Following this, Xu et al. (2021) introduced a multi-threshold LIF model and proved that time compression could be implemented directly during the training process to provide consistency between training and inference. From an algorithmic standpoint, this work investigates multiplicity from the perspective of discretization issues. After taking discrete limitations and membrane potential dynamics into account, we propose Multiple-Spike Pattern (MSP) to permit multiple spike transmissions in a minimal iterative step length [*S*(*t*) in Figure 1], hence making the SNN resistant to varying timescales. MSP enhances the underlying LIF neuron model of SNN algorithms and can be merged easily with a variety of SNN training techniques. Adaptability relates to the Spike Frequency Adaption (SFA) phenomenon of biological neurons, which is highly practical at the algorithmic level (Bellec et al., 2018; Salaj et al., 2021). The benefit of SFA is that it can effectively decrease the number of spikes by temporarily increasing the threshold; hence, we suggest an SFA-based MSP implementation, SFA mode, to minimize spike transmission [*V*(*t*) in Figure 1]. Plasticity refers to the trainability and adjustability of synaptic structures and encourages us to reconsider the dynamical model of synapses. Similar studies have shown that the insertion of the spike response in synapses with plasticity could significantly increase the temporal dependency of the network, resulting in improved performance on spatio-temporal tasks (Jin et al., 2018; Shrestha and Orchard, 2018; Gu et al., 2019; Fang et al., 2020). Nonetheless, the spike response model in synapses explicitly imposes computing costs. The insertion of synaptic state variables to mimic synaptic dynamics is the most poignant step since it necessitates recursive calculations in the time dimension, which are pretty unfriendly for SNN simulation at the algorithmic level. Therefore, we propose a state-free synaptic response model [SFSRM, *O*(*t*) in Figure 1] that does not rely on time-varying states to construct spike response models, which can be implemented using efficient convolution operations as opposed to iterative processes, hence facilitating network training significantly. Experiments have also shown that stateless synaptic responses with plasticity help expedite network convergence and improve performance.

We construct controlled experiments for the proposed models at different discretization levels, according to our mathematical assumptions and constraints, and examine their stability issues under various parameter settings. We evaluate the proposed model using the N-MNIST and SHD neuromorphic datasets (Orchard et al., 2015; Cramer et al., 2020). The experimental results demonstrate that the proposed model can achieve competitive performance on both the N-MNIST and SHD datasets. In addition, comparative and analytical studies reveal that the proposed modeloffers more robust model stability over a range of iterative step lengths, fewer and efficientspike activities, and superior model performance for temporal feature extraction in neuromorphic tasks.

Our main contributions are four-folds:

1. This research highlights the disparity between discrete iterative simulation and biological neural dynamics. In addition, to the best of our knowledge, this study investigates for the first time the discretization problem in time-iteration and introduces a new point about the model's robustness under varying iterative step lengths for BP-based SNN algorithms.
2. Starting with the neuron dynamics of LIF, the analytical model, standard numerical model, and simplified numerical model are derived from the top down. Additionally, this study investigates the constraints of the existing discrete LIF models and extends them from the bottom up. Through mathematical analysis, all the discrete models mentioned are connected in series through constraints.
3. This paper offers the Multiple-Spike Pattern for robust iterative training in Spiking Neural Networks, offering a promising avenue for future SNN algorithm development. In addition, we propose an SFA mode as an MSP implementation for efficient spike activities.
4. This work utilizes a State-Free Synaptic Response Model (SFSRM) to mimic synaptic dynamics for temporal expressiveness, which replaces loop calculation by convolution operation, compatible with deep learning frameworks.

## 2. Methods and materials

### 2.1. The networks components and training methods in spiking neural networks

#### 2.1.1. LIF model as spiking neurons

Dendrites, soma, and axons are the three core constituents of the biological neuronal structure (Zhang et al., 2021). Dendrites are responsible for receiving input signals from pre-synaptic neurons and integrating them into the soma. As the computational hub of the neuron, the soma takes input signals, changes the membrane potential, and generates an action potential (spike activity) when the membrane potential exceeds a threshold. Spikes are conveyed through axons to axonal terminals and serve as input signals to post-synaptic neurons. Based on the neural dynamics, neuroscientists have developed many neural models, represented by the Hodgkin-Huxley (Hodgkin et al., 1952), the leaky integrate-and-fire (LIF) (Dayan and Abbott, 2005), and the Izhikevich (Izhikevich et al., 2004) model. As a simplified model of neuron dynamics, LIF has garnered a great deal of interest from algorithm researchers and has been extensively implemented in SNNs (Lee et al., 2016; Wu et al., 2018; Cheng et al., 2020; Yin et al., 2020; Fang et al., 2021). The LIF model captures the intuitive properties of external input accumulating charge across a leaky neural membrane with a clear threshold (Tavanaei et al., 2019). The LIF model stimulates the accumulation, leakage, and excitation of the membrane potential with the mathematical model as:


(1) $$\begin{array}{l} \tau \frac{dv}{dt}=-[v-V_{rest}]+RI\left(t\right) \end{array}$$

Here τ is the neuron soma's time constant, which equals the product of the capacitance constant *C* and resistance constant *R* of the neural membrane. *I*(*t*) is the overall pre-synaptic input current and is accumulated into the membrane potential *v*(*t*). *V*_*rest*_ is the resting potential of neuron soma. When *v*(*t*) is in the dynamic range (*v*(*t*) ≤ *V*_*th*_), the neuron activity follows the Equation (1), accumulating the membrane potential among time. Once the *v*(*t*) exceeds the potential threshold *V*_*th*_, the neuron fires a spike and resets the membrane potential *v* to *V*_*rest*_ waiting for accumulation again. The generation of the neuron spike activity *s* is defined as:


(2) $$\begin{array}{l} s=g\left(v\right)=\{\begin{array}{c} 0,\;v<V_{th}\\ 1,\;v=V_{th} \end{array} \end{array}$$

The LIF model gives a decent neuron prototype at the algorithmic level that merits additional research. Due to the universality of the LIF model, we use it as the spiking neuron model in our research.

#### 2.1.2. Learning rules in SNNs

In SNNs, synaptic strengths are described as scalar weights that can be dynamically modified according to a particular learning rule. Actively investigated, the learning rules of SNNs can be generally categorized into three categories: conversion-based methods that map SNNs from trained ANNs (Diehl et al., 2016; Hunsberger and Eliasmith, 2016; Rueckauer et al., 2016, 2017; Sengupta et al., 2019; Han et al., 2020); supervised learning with spikes that directly train SNNs using variations of error backpropagation (Lee et al., 2016; Shrestha and Orchard, 2018; Wu et al., 2018, 2019; Neftci et al., 2019; Yin et al., 2020; Fang et al., 2021); local learning rules at synapses, such as schemes exploring the spike time dependent plasticity (STDP) (Song et al., 2000; Nessler et al., 2009; Diehl and Cook, 2015; Tavanaei et al., 2016; Masquelier and Kheradpisheh, 2018). In addition to the above-mentioned directions, many new algorithms have emerged, such as: a biological plausible BP implementation in pyramidal neurons based on the Bursting mechanism (Payeur et al., 2021); a biologically plausible online learning based on rewards and eligibility traces (Bellec et al., 2020); and the target-based learning in recurrent spiking networks (Ingrosso and Abbott, 2019; Muratore et al., 2021), which provides an alternative to error-based approaches.

By defining pseudo-derivatives for non-differentiable spike activity, recent research has successfully adapted the backpropagation technique to SNNs (Lee et al., 2016; Wu et al., 2018; Tavanaei et al., 2019; Cheng et al., 2020). These BP-based SNNs are comparable to extensions of conventional Recurrent Neural Networks (RNNs) that use error backpropagation *via* time and gradient descent to update connection weights. The BP-based algorithms can leverage mature deep learning frameworks for network design and operational efficiency, and have become an essential branch of algorithmic developments. Consequently, this study will use the basic surrogate technique in BP-SNNs to train the proposed model.

#### 2.1.3. Synaptic dynamics in BP-based SNNs

Following in the footsteps of neuromorphic computing, Synaptic Dynamics research is being investigated. Some research that applied bio-inspired Synaptic Models in BP-SNNs exhibited improved performance, such as Lateral Interactions for intra-layer connections (Cheng et al., 2020) and the Spike Response Model (SRM) for synaptic expressions (Jin et al., 2018; Shrestha and Orchard, 2018; Gu et al., 2019; Fang et al., 2020), providing a starting point for embedding synaptic dynamics into deep-SNNs.

### 2.2. Multiplicity with multiple-spike pattern

#### 2.2.1. Iterable LIF model with single-spike pattern

To represent the SNN at the algorithmic level, the computational links between the specified variables must be shown. The LIF differential definition (Equation 1) must thus be rewritten in discrete form. Let *v*_*rest*_ = 0, Equation (1) yields the following expression:


(3) $$\begin{array}{l} v\left(t\right)=e^{-\frac{t_{k}}{\tau }}\times v\left(t-t_{k}\right)+\frac{R}{\tau }\int_{0}^{t_{k}}e^{-\frac{\tau_{d}}{\tau }}\times I\left(t-\tau_{d}\right)d\tau_{d} \end{array}$$

where *t*_*k*_ indicates the time difference, and *v*(*t*) is obtained by adding two factors, namely its own attenuation and the input response.

An explicitly iterative version of the LIF model is generally utilized in deep learning frameworks (Wu et al., 2018; Cheng et al., 2020), allowing discrete neural spike activities in deep SNNs. From Equation (3), by establishing the minimal iterative step size Δ*t*, the following discrete iterative model of LIF is obtained:


(4) $$\begin{array}{l} v\left[t\right]=e^{-\frac{\Delta t}{\tau }}\times v\left[t-\Delta t\right]+\left(1-e^{-\frac{\Delta t}{\tau }}\right)\times RI\left[t\right] \end{array}$$

where *I*[*t*] indicates the current input at time *t*. The model assumes that the current is consistent throughout the time interval (*t* − Δ*t, t*] with a magnitude of *I*[*t*]. This assumption inevitably results in the quantization error (as shown in the **Figure 3**). Theoretically, when Δ*t* → 0, the quantization error approaches zero.

Alternatively, considering computational simplification, we have:


(5) $$\begin{array}{l} v\left[t\right]=\tau_{decay}\times v\left[t-\Delta t\right]+Ĩ\left[t\right] \end{array}$$

Here τ_*decay*_ is the time constant describing the leaky activity of LIF model, which equals to $e^{-\frac{\Delta t}{\tau }}$. Ĩ[*t*] is the normalized pre-synaptic input current, which equals to $\left(1-e^{-\frac{\Delta t}{\tau }}\right)RI\left[t\right]$. In the discrete simulation, *s*[*t*] represents the spike activity during the time interval (*t* − Δ*t, t*], defined as:


(6) $$\begin{array}{l} s\left[t\right]=g\left(v\left[t\right]\right)=\{\begin{array}{c} 0,\;v\left[t\right]<V_{th}\\ 1,\;v\left[t\right]\geq V_{th} \end{array} \end{array}$$

By unifying the accumulation activity and spike activity, the hard-reset LIF model (Wu et al., 2018; Eshraghian et al., 2021) for discrete computation becomes:


(7) $$\begin{array}{l} v[t]=\tau_{decay}\times v[t-\Delta t]\times (1-s[t-\Delta t])+\tilde{I}[t] \end{array}$$

A crucial assumption of the LIF iterative discrete model is Δ*t* → 0, which guarantees: 1. The input term Ĩ[*t*] is sufficiently small such that *sup*{*v*[*t*]} ≈ *V*_*th*_; 2. In the span of time (*t* − Δ*t, t*], only one spike may fire. Under these conditions, the hard-reset LIF model effectively matches neuronal dynamics (Equation 1).

**Figure 2:**
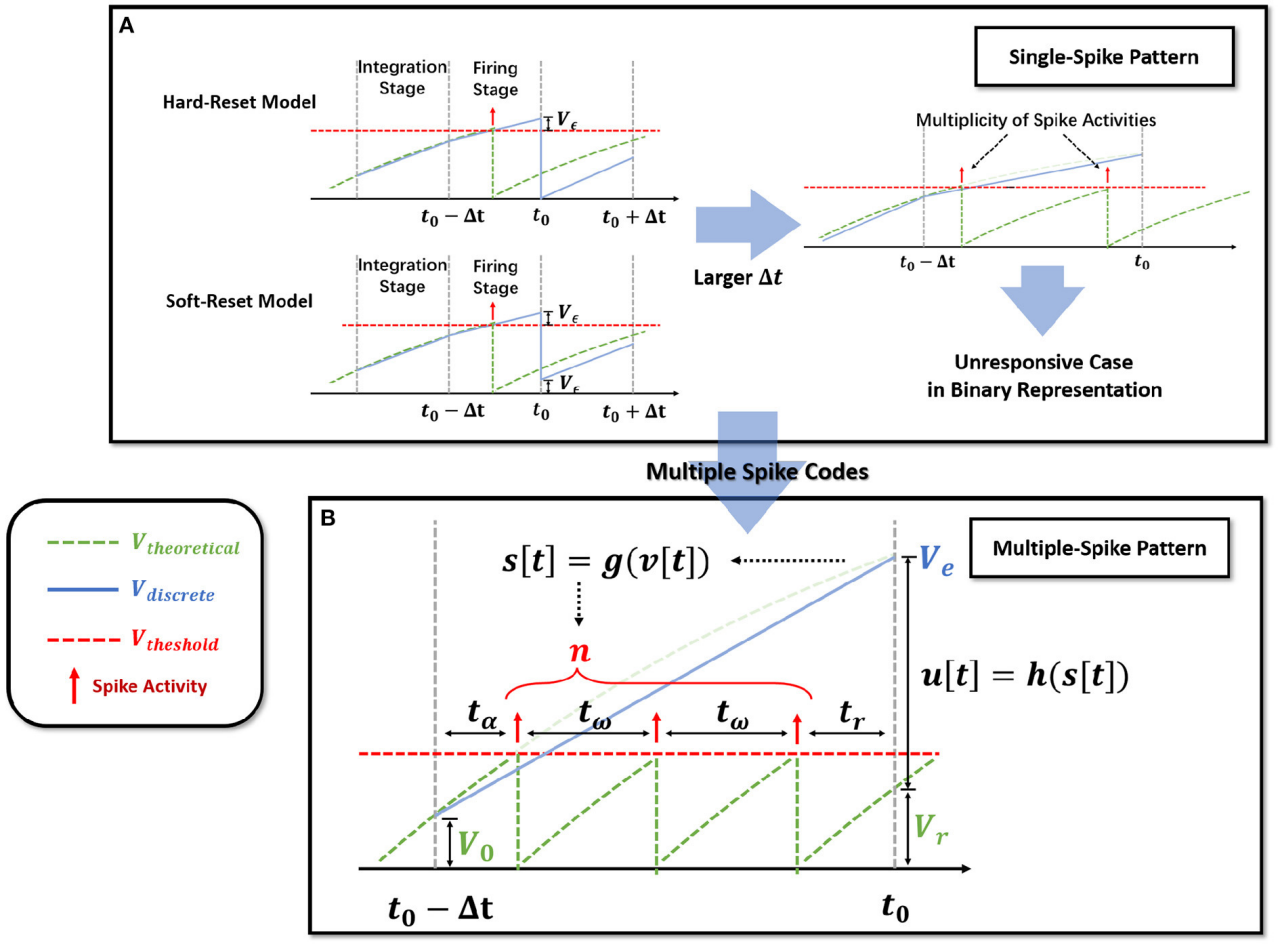
Extension of the single-spike pattern (SSP) to multiple-spike pattern (MSP). **(A)** Modeling LIF neural dynamics with Hard-Reset Model and Soft-Reset Model, which derives the multiplicity issue with larger Δ*t*. **(B)** Extending the SSP into MSP with integer transmission, which estimates the number of spike activities inside a Δ*t* time span.

Notably, observe that in the hard-reset version, *sup*{*v*[*t*]} > *V*_*th*_ is always present. Assuming *v*[*t*_0_]−*V*_*th*_ = *V*_ϵ_[*t*_0_] > 0 at *t*_0_, hard-reset resets the membrane potential straight to zero and disregards the membrane potential difference *V*_ϵ_[*t*_0_], as shown in Figure 2A. It is reasonable because when Δ*t* is sufficiently small, *V*_ϵ_[*t*_0_] → 0.

The soft-reset model is introduced to keep the portion of *V*_ϵ_ that exceeds the threshold (Figure 2A), and its expression is as follows:


(8) $$\begin{array}{l} v[t]=\tau_{decay}\times (v[t-\Delta t]-s[t-\Delta t]\times V_{th})+\tilde{I}[t] \end{array}$$

Soft-reset is based on the claim that the membrane potential *v*_ϵ_ beyond the threshold contains implicit information that must be retained, which is often employed in the ANN-SNN conversion technique (Han et al., 2020). However, as shown in Figure 2A, the *v*_ϵ_[*t*_0_] produced *via* the soft-reset approach remains distinct from the theoretical value *V*_*theoretical*_(*t*_0_). In Section 2.2.3, we will demonstrate that when $\frac{\Delta t}{\tau }\to 0$ and $\frac{V_{th}}{RI\left[t\right]}\to 0$, *v*_ϵ_[*t*_0_] = *V*_*theoretical*_(*t*_0_) holds.

Iterable neural models applied to time-iteration work similarly to recurrent neural model, which is discretized into minimal iterative step length Δ*t*. Nevertheless, the difference is that the two common iterable LIF models, the hard-reset model and the soft-reset model, only rely on binary signals to indicate whether a spike activity is generated or not. To differentiate, we refer to discrete models that transmit binary digits (the hard-reset model and the soft-reset model) as Single-Spike Pattern (SSP).

#### 2.2.2. Extending single-spike pattern to multiple-spike pattern

BP-based SNNs simulate the spike activities by discrete time-iteration. Nonetheless, the discreteness presents significant issues with spike multiplicity (Figure 2A). Δ*t* must be carefully set as the minimal iterative step length for simulation inside recursive time-iterations in order to reduce the mismatch between brain dynamics and its discrete behavior.

It should be noted that when Δ*t* begins to rise, the input term coefficient $\left(1-e\left(-\frac{\Delta t}{\tau }\right)\right)$ rises as well, raising $\tilde{I\left[t\right]}$. Two presumptions, at most one spike firing in each time period, and the occurrence of *sup*{*v*[*t*]} ≈ *V*_*th*_, may be erroneous when Δ*t* is large. Since models with SSP represent spike activities as binary sequences, only one spike activity can be handled per iterative step. Under this circumstance, the temporal feature is restricted with lost spikes. Therefore, in discrete time-iteration, the proper selection of the iterative step lengths is always an indispensable part of SSP.

To address the issue of spike loss and lessen the discrepancy between discrete models and neuron dynamics, the idea of Multiple-Spike-Pattern (MSP) is developed, where the number of spikes in (*t* − Δ*t, t*] is permitted to be greater than one, relaxing the restrictions of discrete iterative models and improving the model's stability under various Δ*t* choices. Multiple-Spike Pattern relies on the integer digits transmission, which requires neural models to produce countable spike activities during a minimal time window. Spiking neurons defined with MSP can be represented mathematically as follow:


(9) $$\begin{array}{l} v[t]=\tau_{decay}\times (v[t-\Delta t]-u[t-\Delta t])+\tilde{I}[t] \end{array}$$

(10) $$\begin{array}{l} s\left[t\right]=g\left(v\left[t\right]\right)\;u\left[t\right]=h\left(s\left[t\right]\right) \end{array}$$

**Figure 3:**
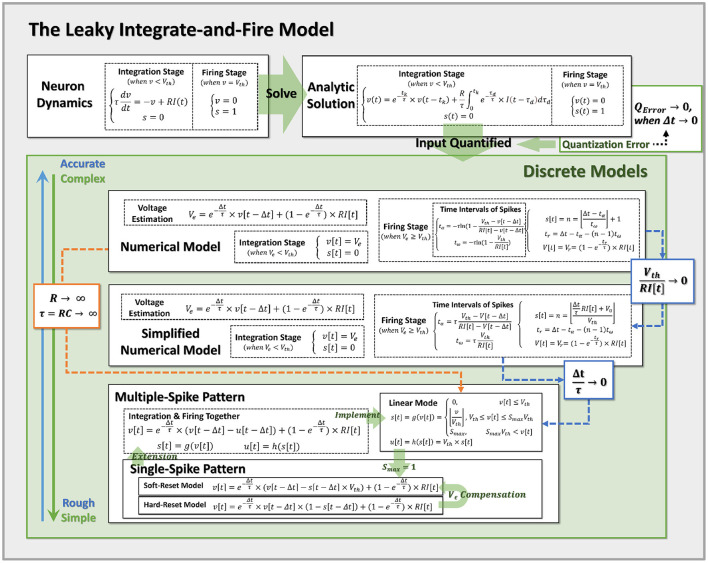
Pipeline for discretization from neuron dynamics to discrete models of varying complexity. By incorporating the prior assumptions, we infer the neural dynamics from top to bottom and derive the more reduced discrete models with the approximation conditions. Notably, Hard-Reset Model and Soft-Reset Model are positioned at the bottom of the discrete model, implying they are the most basic and simplest models.

Here, the spike activities *s*[*t*] is determined by membrane potential *v*[*t*] as integer numbers. *u*[*t*] is the consumed membrane potential that produces multiple spike activities, determined by *s*[*t*]. The implementation of MSP neurons is flexible, that can be achieved with different *g*(·) and *h*(·), to represent different neural dynamics. The definition of MSP neurons is similar with soft-reset SSP (Equation 8), where *u*[*t* − Δ*t*] corresponds to *s*[*t* − Δ*t*] × *V*_*th*_, representing the cost of membrane potential for spike activities. It can be shown that the membrane potential dynamics of SSP (Equations 7, 8) and MSP (Equations 9, 10) are extremely similar. The only difference between SSP and MSP is that MSP redefines the computation of the firing stage, including spike number *s*(·) and cost membrane potential *g*(·). The maximum number of spikes allowed by SSP is 1, but the maximum number of spikes permitted by MSP is more than 1. As shown in Figure 3, the SSP, including both the soft-reset and hard-reset models, could be regarded as the special implementation of the MSP.

The above-described MSP is quite simple and scalable, allowing it to directly replace the LIF model in the BP-SNN. Similar to our MSP, Xu et al. (2021) used a multi-threshold neuron model to achieve SNNs with ultra-low latency, demonstrating the feasibility of multiplicity in compressing features. They provide a suitable MSP implementation, which we refer to as Linear mode, defined as:


(11) $$\begin{array}{l} s\left[t\right]=g\left(v\left[t\right]\right)=\{\begin{array}{cc} 0, & \;v\left[t\right]<V_{th}\\ \left\lfloor \frac{v\left[t\right]}{V_{th}}\right\rfloor , & \;V_{th}\leq v\left[t\right]<S_{max}V_{th}\\ {\;S}_{max}, & \;S_{max}V_{th}\leq v\left[t\right] \end{array} \end{array}$$

(12) $$\begin{array}{l} u\left[t\right]=h\left(s\left[t\right]\right)=V_{th}\times s\left[t\right] \end{array}$$

**Figure 4:**
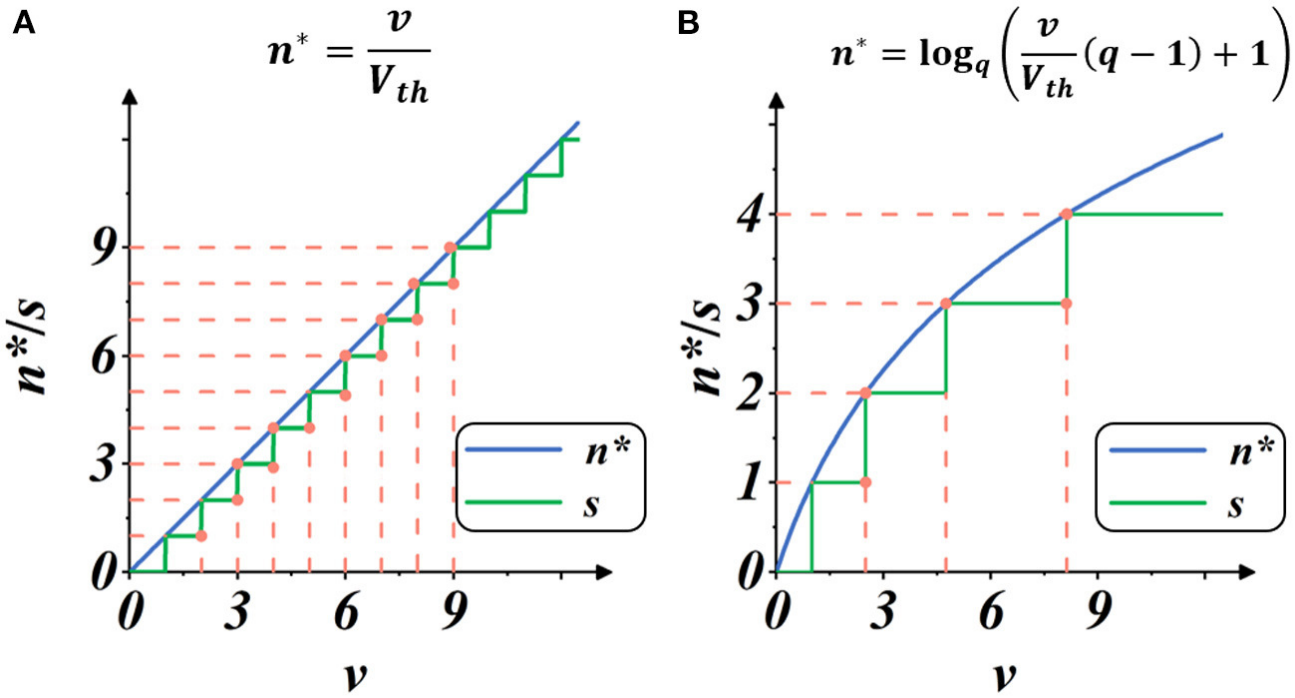
Plots of spike gate function *s* = *g*(*v*) (Equation 10): the spike activity vs. membrane potential. The blue plot indicates the continuous estimate of the spike intensity n*, and the green plot indicates the discrete count of the spike activities *s*. The threshold is set as *V*_*th*_ = 1. **(A)** Spike activities in Linear mode. **(B)** Spike activities in Spike Frequency Adaption mode with *q* = 1.5.

Here, *S*_*max*_ is the maximum integer value of *g*(·), representing the upper limit of spike activities within a certain iterative state. We regard the implementation as Linear mode because it provided a linear estimate of spike intensity, as shown in Figure 4A.

#### 2.2.3. From neural dynamics to multiple-spike pattern

Now, we return to the differential model and re-examine the discretization method of MSP from the standpoint of theoretical calculation. First, it must be made clear that we still require the input current in the discrete window to be constant (corresponding to Input Quantified in Figure 3), and let *I*_*c*_ = *I*[*t*_0_], which means that the current input to the neuron at the time *t*_0_ is used as the assumed constant in the time of (*t*_0_−Δ*t, t*_0_]. There is no way to avoid the quantization error of the current during discretization, but fortunately, when Δ*t* → 0, the quantization error approaches zero.

Theoretically, the firing time interval of the LIF model is consistent under a constant current; consequently, we establish the process of membrane potential change and spike activity with the spike time interval inside the Δ*t* window. As shown in Figure 2B, let *t*_α_ represent the time necessary to fire the initial spike, *t*_ω_ represent the time between successive pulses, and *t*_*r*_ represents the time difference between the final spike and *t*_0_. According to the neuron parameters τ, *R*, the input current *I*_*c*_, and the membrane potential *V*_0_ = *v*[*t*_0_−Δ*t*], the number of spike *n* and the residual membrane potential *V*_*r*_ = *v*[*t*_0_] can be calculated.

The specific computation procedure is as described below. First, according to the differential definition (Equation 1), the estimated potential value *V*_*e*_ at time *t*_0_ is solved,


(13) $$\begin{array}{l} V_{e}=e^{-\frac{\Delta t}{\tau }}V_{0}+\left(1-e^{-\frac{\Delta t}{\tau }}\right)RI_{c} \end{array}$$

In the case of *V*_*e*_<*V*_*th*_, the potential cannot be charged to the threshold, and no spike activity is generated (so-called Integration Stage in Figure 3), at this moment:


(14) $$\begin{array}{l} \{\begin{array}{l} n=0\\ V_{r}=V_{e}=e^{-\frac{\Delta t}{\tau }}V_{0}+\left(1-e^{-\frac{\Delta t}{\tau }}\right)RI_{c} \end{array} \end{array}$$

When *V*_*e*_ ≥ *V*_*th*_, there exists spike activities (*n* ≥ 1) within window. In this case, the two criteria *RI*_*c*_ > *V*_*th*_ and *t*_α_ ≤ Δ*t* are satisfied. The following equations are determined mathematically:


(15) $$\begin{array}{l} \{\begin{array}{l} t_{\alpha }=-\tau ln\left(1-\frac{V_{th}-V_{0}}{RI_{c}-V_{0}}\right)\\ t_{\omega }=-\tau ln\left(1-\frac{V_{th}}{RI_{c}}\right)\\ n=\left\lfloor \frac{\Delta t-t_{\alpha }}{t_{\omega }}\right\rfloor +1\\ t_{r}=\Delta t-t_{\alpha }-\left(n-1\right)t_{\omega }\\ V_{r}=\left(1-e^{-\frac{t_{r}}{\tau }}\right){RI}_{c} \end{array} \end{array}$$

Note that if $\frac{V_{th}}{RI_{c}}\to 0$, the calculation of case *V*_*e*_ ≥ *V*_*th*_ could be simplified:


(16) $$\begin{array}{l} \{\begin{array}{l} t_{\alpha }=-\tau ln\left(1-\frac{V_{th}-V_{0}}{RI_{c}-V_{0}}\right)\approx \tau \frac{V_{th}-V_{0}}{RI_{c}-V_{0}}\\ t_{\omega }=-\tau \ln\left(1-\frac{V_{th}}{RI_{c}}\right)\approx \tau \frac{V_{th}}{RI_{c}}\\ n=\left\lfloor \frac{\Delta t-t_{\alpha }}{t_{\omega }}\right\rfloor +1\\ \approx \left\lfloor \frac{\Delta t-\tau \frac{V_{th}-V_{0}}{RI_{c}-V_{0}}}{\tau \frac{V_{th}}{RI_{c}}}\right\rfloor +1\\ \approx \left\lfloor \frac{\frac{\Delta t}{\tau }RI_{c}+V_{0}}{V_{th}}\right\rfloor \\ t_{r}=\Delta t-t_{\alpha }-\left(n-1\right)t_{\omega }\\ V_{r}=\left(1-e^{-\frac{t_{r}}{\tau }}\right){RI}_{c} \end{array} \end{array}$$

Through the above-mentioned standard numerical model and simplified numerical model, we are able to construct the LIF model under discrete circumstances, whose membrane potential change and number of spike activities closely resemble the neuron dynamics under quantified input.

Regarding the unconstrained Linear mode (Equation 11) with *S*_*max*_ → ∞), it can be shown that if the condition $\frac{\Delta t}{\tau }\to 0$ is met, the Linear mode can achieve the same number of spikes as the simplified analytic model (Equation 16):


(17) $$\begin{array}{l} \begin{array}{l} s\left[t_{0}\right]=g\left(v\left[t_{0}\right]\right)=\left\lfloor \frac{V_{e}}{V_{th}}\right\rfloor \\ =\left\lfloor \frac{{e^{-\frac{\Delta t}{\tau }}V}_{0}+\left(1-e^{-\frac{\Delta t}{\tau }}\right){RI}_{c}}{V_{th}}\right\rfloor \\ \approx \left\lfloor \frac{\left(1-\frac{\Delta t}{\tau }\right)V_{0}+\frac{\Delta t}{\tau }{RI}_{c}}{V_{th}}\right\rfloor \\ \approx \left\lfloor \frac{V_{0}+\frac{\Delta t}{\tau }{RI}_{c}}{V_{th}}\right\rfloor =n \end{array} \end{array}$$

At this time, considering the reset of membrane potential,


(18) $$\begin{array}{l} V_{\epsilon }\left[t_{0}\right]=v\left[t_{0}\right]-u\left[t_{0}\right]=V_{e}-n\times V_{th}\\ ={e^{-\frac{\Delta t}{\tau }}V}_{0}+\left(1-e^{-\frac{\Delta t}{\tau }}\right){RI}_{c}-\left\lfloor \frac{\Delta t}{\tau }RI_{c}+V_{0}\right\rfloor \\ \approx V_{0}+\frac{\Delta t}{\tau }RI_{c}-\left\lfloor \frac{\Delta t}{\tau }RI_{c}+V_{0}\right\rfloor =\frac{t_{r}}{\tau }RI_{c}\\ \approx \left(1-e^{-\frac{t_{r}}{\tau }}\right){RI}_{c}=V_{r} \end{array}$$

Accordingly, when the condition $\frac{\Delta t}{\tau }\to 0$ is met, we demonstrate that the linear mode is an approximation of the simplified analytical model. Also, we may easily switch the standard numerical model to the linear mode by adjusting *R* → ∞, and τ = *RC* → ∞ (while keeping the conductance coefficient $C=\frac{\tau }{R}$ constant), at which point the membrane potential will no longer decrease and the LIF model will degenerate into the IF model. All obtained models and their approximation relations are summarized in Figure 3, which simplifies the top-down modeling of neuron dynamics. Notably, Hard-Reset Model and Soft-Reset Model are positioned at the bottom of the discrete model, implying they are the most basic and simplest models.

### 2.3. Multiple-spike pattern with adaptability

#### 2.3.1. Spike frequency adaptation for spike activities

Spike-frequency adaptation (SFA) is a biological neural phenomenon describing a neuron fires with a frequency that reduces over time when stimulated with constant input. The phenomenon occurs in both vertebrates and invertebrates, in peripheral and central neurons, and plays an essential role in neural information processing (Benda and Herz, 2003). The SFA mechanism leads to non-linearity in spike activities and enriches the temporal feature for a single spike. Specifically, Adibi et al. (2013) suggest that the SFA mechanism in real neurons like whisker sensory cortexes helps improve the information capacity of a single spike defined by the average mutual information (MI). Therefore, this work adopts the SFA mechanism with MSP to improve spike transmission efficiency.

#### 2.3.2. MSP implementation with SFA

Under the SFA mechanism, the threshold of the neural model will be temporarily raised when a spike occurs to suppress the excitement of dense spike activities.


(19) $$\begin{array}{l} V_{th}\left[s_{n+1}\right]=q\times V_{th}\left[s_{n}\right] \end{array}$$

Here *V*_*th*_[*s*_*n*_] indicates the membrane threshold when *n*^*th*^ spike activity occurs, *q* is the inhibition coefficient used to control the temporary raising of *V*_*th*_, making the intensity of spike activity drop exponentially.

In this case, the required membrane potential *u* to generate *s* times of spike activities is given by the sum of geometric sequence with the neural threshold basis *V*_*th*_:


(20) $$\begin{array}{l} u=\overset{s}{\underset{i=1}{\sum }}\left(q^{i}\times V_{th}\right)=\frac{q^{s}-1}{q-1}\times V_{th} \end{array}$$

Similarly, we can directly estimate the value of spike activities through analytic expressions:


(21) $$\begin{array}{l} v=\frac{q^{n^{*}}-1}{q-1}\times V_{th} \end{array}$$

Equivalently,


(22) $$\begin{array}{l} n^{*}=\log_{q}\left[\frac{v}{V_{th}}\left(q-1\right)+1\right] \end{array}$$

Here n^*^ is the estimated intense spike activity. Then, the exact value of spike activities *s* is given by:


(23) $$\begin{array}{l} s=\left\lfloor n^{*}\right\rfloor \end{array}$$

On this basis, *g*(·) and *h*(·) in Equation (10) are clearly defined, which shows that the SFA implementation under MSP can be directly determined without step-by-step calculation.

As shown in Figure 4B, when the membrane potential *v* increases, the intensity of spike activity gradually deviates from linearity, showing adaptability to the current input. In this case, the total number of spike activities decreases, and each spike activity brings more features, potentially saving computation operations with less spike transmission while maintaining high performance.

In order to apply backpropagation, we assign a particular pseudo derivative as follows:


(24) $$\begin{array}{l} \frac{\partial s}{\partial n^{*}}=1 \end{array}$$

This pseudo-derivative provides a unit vector for gradient descent without complicated computations.

### 2.4. Plasticity with state-free synaptic response model

#### 2.4.1. Modeling spike activity through synaptic dynamics

In biological neural networks with synaptic dynamics, a spike is thought to originate in the soma, travel up the axon to the synapse, and then be transformed into an electric current in the dendrites of the connected neuron. Spike Response Model (SRM) is presented for describing synaptic dynamics by transforming spike activity into current response flowing into post-synaptic dendrites (Gerstner and Kistler, 2002), defined as:


(25) $$\begin{array}{l} o\left(t\right)=f\left(s,t\right)=\left(K*s\right)\left(t\right) \end{array}$$

Here *s*(*t*) is the spike activities, *o*(*t*) is the spike response signal transmitted from axon terminal to dendrite over time, *K*(*t*) is the spike response kernel relating current intensities with spike activities. The incoming spikes *s*(*t*) is converted into spike response signal *o*(*t*) by convolving with a spike response kernel *K*(·). General expressions for spike response kernel *K*(*t*) are 1- and 2-exponential functions (Rothman, 2013), such as the following:


(26) $$\begin{array}{l} K\left(t\right)=e^{-t/\tau_{d}} \end{array}$$

(27) $$\begin{array}{l} K\left(t\right)=e^{-t/\tau_{d}}-e^{-t/\tau_{r}} \end{array}$$

Here τ_*r*_ and τ_*d*_ are the rise and decay time constants. The disadvantage of the simple 1-exponential ignores the finite rise time of the synaptic conductance, rising instantaneously from 0 to 1. Hence, the 2-exponential function is used in the proposed model since it contains a finite rise time.

By discretizing the convolutional operation, the spike response signal *o*(*t*) can be represented as:


(28) $$\begin{array}{l} o\left(t\right)=\overset{i\cdot \Delta t\leq t}{\underset{i=0}{\sum }}s\left(t-i\cdot \Delta t\right)K\left(i\cdot \Delta t\right) \end{array}$$

The dendrites synthesize the input current intensity from all connected axon terminals and act directly on the membrane potential state of the neuron at some certain time *t* as Equation (29).


(29) $$\begin{array}{l} I_{i}\left(t\right)=\underset{j}{\sum }w_{ij}o_{j}\left(t\right)+bias_{i}\left(t\right) \end{array}$$

Here *I*(*t*) is the overall pre-synaptic input current and is accumulated into the membrane potential, *w*_*ij*_ is the synaptic connecting weight from neuron *j* to neuron *i* and *bias*_*i*_ is the constant background input.

#### 2.4.2. Potential plasticity in spike response model (SRM)

**Figure 5:**
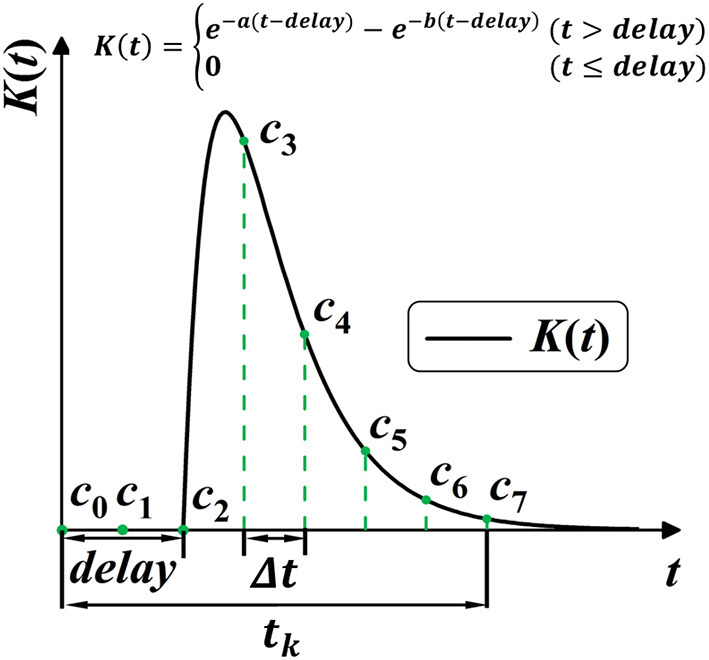
Model the SRM *K*(*t*) into 1-D convolution kernel *C*_*n*_. *K*(*t*) is modeled with three trainable parameters, with *a*, *b* for shape, and *delay* for the time delay.

SRM provides richer temporal information for the network by allowing the varying effect of certain spike activity. However, the constant parameters of response kernel *K*(*t*) are widely pre-defined as a “ground truth” before training, which limits the potential diversity and plasticity for SRM. Shrestha and Orchard (2018) first considered the plasticity of SRM by setting response delay as learnable parameters, which unsurprisingly improved the performance. As shown in Figure 5, this work further frees up the shape parameters for better plasticity, allowing shape parameters *a*, *b*, and delay parameter *delay* to be learnable during training. In this case, the plasticity of spike activity allows each neuron to learn different temporal features, improving the complexity and fitting ability of the model.

#### 2.4.3. Spike response model as 1-D convolution

Common techniques for implementing synaptic dynamics and SRM require indicating state variables inside the synapse for generating post-synaptic currents through time iterations (Jin et al., 2018; Gu et al., 2019; Fang et al., 2020). As shown in Figure 5, SRMs used for spike activities are going up to the peak and subsequently decreasing toward zero (Rothman, 2013). We observe that it is feasible to minimize computing complexity when constructing a state-free synaptic response model (SFSRM) by omitting the long-term spike response. This work applies one-dimensional convolution operation in computing SRM by defining a valid time window *t*_*k*_ to make the SRM more efficient and more compatible with nowadays deep learning frameworks. The convolution operation follows as:


(30) $$\begin{array}{l} o\left(t\right)=f\left(s,t\right)=\overset{i\cdot \Delta t\leq t_{k}}{\underset{i=0}{\sum }}s\left(t-i\cdot \Delta t\right)\times C_{i} \end{array}$$

Here *C*_*n*_ is the one-dimensional convolutional kernel of spike responses, modeled with three variables *a*, *b* and *decay*. *t*_*k*_ is the time-window constant that describes the necessary scope of spike response, defined as Equation (31) with the minimal iterative step length Δ*t* and convolution kernel size.


(31) $$\begin{array}{l} t_{k}=\left(kernel\text{\_}size-1\right)\times \Delta t \end{array}$$

Then, as shown in Figure 5, the discrete self-learning *C*_*n*_ works as the delayed spike response kernel *K*(*t*). According to the 2-exponential kernel in Equation (27), we model the discrete convolutional kernel *C*_*n*_ by delay parameter *delay*, and two shape parameters *a* and *b*, as:


(32) $$\begin{array}{l} K^{*}\left(t\right)=e^{-at}-e^{-bt} \end{array}$$

(33) $$\begin{array}{l} K\left(t\right)=\{\begin{array}{c} K^{*}\left(t-delay\right),\;t\geq delay\\ 0,\;t<delay \end{array} \end{array}$$

(34) $$\begin{array}{l} C_{i}=K\left(i\;\cdot \text{Δ}t\right) \end{array}$$

Based on the above definition, we can use a simple convolution operation to describe the integration of information in the temporal dimension. The SFSRM is defined as trainable if its three shape parameters are adjusted toward the objective throughout the learning process. The idea of spike plasticity is then linked to the adjustability of the SRM.

### 2.5. The model dataflow overview

**Figure 6:**
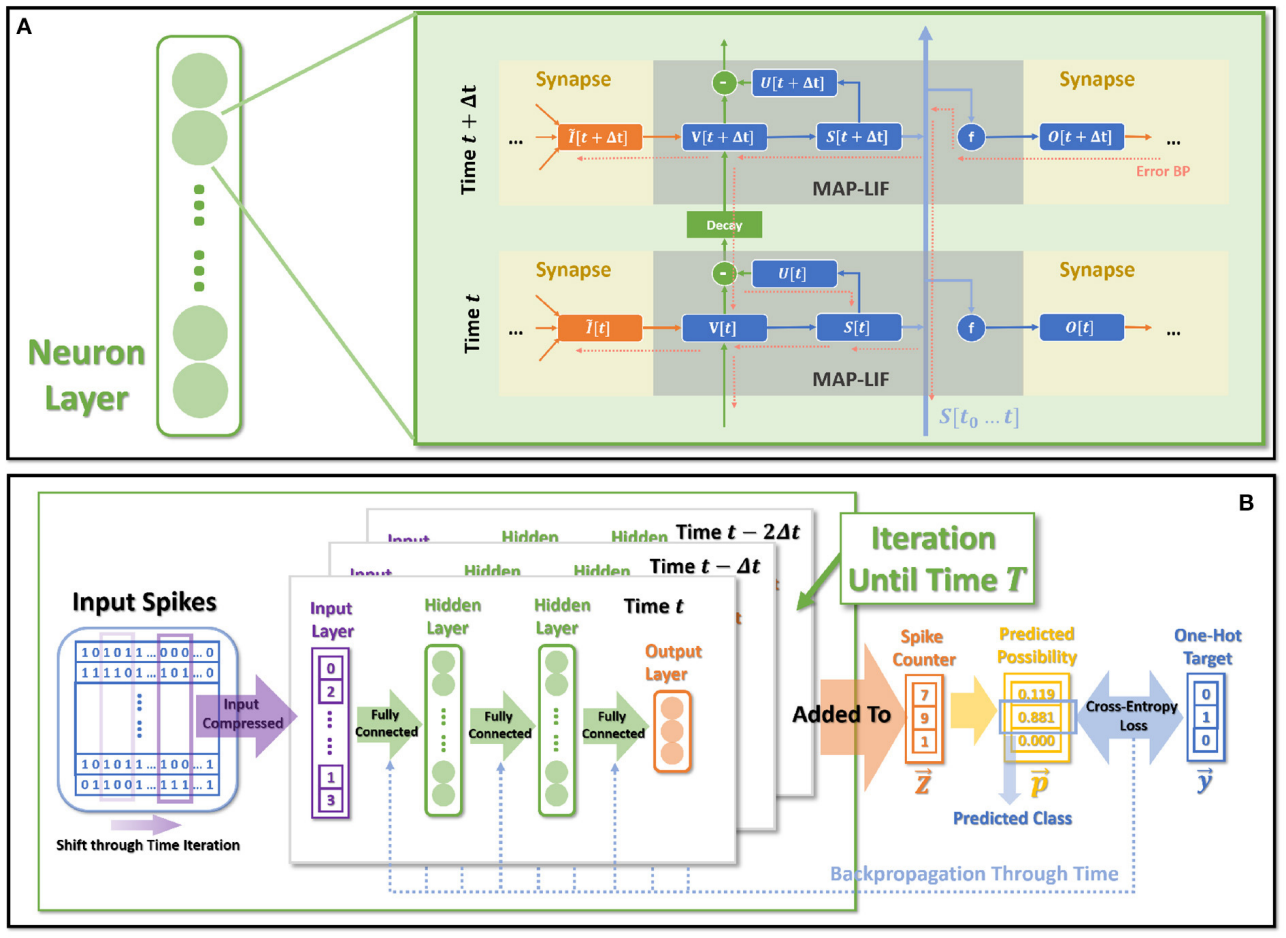
The overall model dataflow. **(A)** The spatio-temporal feedforward and backward dataflow in the proposed model. Each box of MAP-LIF indicates a certain spatio-temporal iterative state. **(B)** The training process for classification tasks.

Figure 6A shows the spatio-temporal dataflow of the neural model in a certain iterative state. The solid arrows indicate spatial and temporal feedforward; the red dotted arrows indicate the error backpropagation correspondingly along with the reverse directions of feedforward. We list more specific mathematical relationships of each variable below:


(35) $$\begin{array}{l} {Î}_{i}^{n}\left[t\right]=\overset{L^{n}}{\underset{i=1}{\sum }}w_{ij}^{n}O_{j}^{n-1}\left[t\right]+bias_{i}^{n} \end{array}$$

(36) $$\begin{array}{l} V_{i}^{n}[t]=\tau_{decay}\times (V_{i}^{n}[t-\Delta t]-U_{i}^{n}[t-\Delta t])+{\hat{I}}_{i}^{n}[t] \end{array}$$

(37) $$\begin{array}{l} S_{i}^{n}[t]=\left\lfloor \log_{q_{i}^{n}}\left[\frac{V_{i}^{n}[t]}{V_{th_{i}}^{n}}\left(q_{i}^{n}-1\right)+1\right]\right\rfloor \end{array}$$

(38) $$\begin{array}{l} U_{i}^{n}\left[t\right]=\overset{S_{i}^{n}\left[t\right]}{\underset{k=1}{\sum }}\left({\left(q_{i}^{n}\right)}^{k}\times V_{{th}_{i}}^{n}\right)\\ \;=\frac{{\left(q_{i}^{n}\right)}^{S_{i}^{n}\left[t\right]}-1}{q_{i}^{n}-1}\times V_{{th}_{i}}^{n} \end{array}$$

(39) $$\begin{array}{l} O_{j}^{n}[t]=f\left(S_{i}^{n}[t-t_{k}],...S_{i}^{n}[t-\Delta t],S_{i}^{n}[t]\right)\\ \;=\sum_{d=0}^{d\cdot \Delta t\leq t_{k}}\left(S_{i}^{n}[t-d\cdot \Delta t]\times K_{i}^{n}(d\cdot \Delta t)\right) \end{array}$$

(40) $$\begin{array}{l} K_{i}^{n}\left(t\right)=\{\begin{array}{l} e^{-a_{i}^{n}\left(t-delay_{i}^{n}\right)}-e^{-b_{i}^{n}\left(t-delay_{i}^{n}\right)}\;\left(t\geq delay_{i}^{n}\right)\\ 0\;\left(t<delay_{i}^{n}\right) \end{array} \end{array}$$

The explanation of the formulas is as follows:

- The sequences with three indices *n*, *i*, *t* represent states of *i*-th neuron at the *n*-th layer and the *t*-th time point. The variables with two indices *n*, *i* represent learnable parameters of *i*-th neuron at the *n*-th layer. *L*^*n*^ indicates the number of neurons in the *n*-th layer.
- ${Î}_{i}^{n}\left[t\right]$ represents the input weighted summation of the output currents from the previous layer.
- $V_{i}^{n}\left[t\right]$ represents the neuron's membrane potential.
- $S_{i}^{n}\left[t\right]\in Z$ represents the number of spikes activities of the neuron within iterative time (*t* − Δ, *t*].
- $U_{i}^{n}\left[t\right]$ represents the consumed mem-potential used for producing spike activities.
- $O_{i}^{n}\left[t\right]$ represents the output currents of the neuron, which is determined by the past spike activities with the convolution operation *f*(·). The kernel of convolution is defined as $K_{i}^{n}\left(t\right)$, modeled with $delay_{i}^{n}$, $a_{i}^{n}$, $b_{i}^{n}$.
- $V_{{th}_{i}}^{n}$, $q_{i}^{n}$, $a_{i}^{n}$, $b_{i}^{n}$, and $delay_{i}^{n}$ are parameters of *i*-th neuron at the *n*-th layer, as previously described.

### 2.6. Objective and training process

The entire dataflow of the training procedure is shown in Figure 6B. The whole training process is based on BPTT, which derives gradients for certain parameters through error-backpropagation and applies gradients decent for optimization.

Input layer collects all spikes arising during (*t* − Δ*t, t*] as $O_{i}^{0}\left[t\right]$. The $O_{i}^{0}\left[t\right]$ is then fully connected (Equation 35) to the integrated inputs for the first hidden layer, following the dataflow shown by Equations (35)–(40).

Before applying the BP-based SNNs to classification tasks, we need to define the decoding map from the output spikes to the corresponding target labels. Refer to the rate coding, we count the total number of output spikes $\vec{Z}$ and derive the predicted probability distribution of targets $\vec{p}$ based on the softmax of spike counters,


(41) $$\begin{array}{l} Z_{i}=\overset{T}{\underset{t}{\sum }}S_{i}^{out}\left[t\right] \end{array}$$

(42) $$\begin{array}{l} p_{i}=\frac{e^{Z_{i}}}{\overset{L_{out}}{\underset{j}{\sum }}e^{Z_{j}}} \end{array}$$

Here *T* is the total number of simulation timesteps, $S_{i}^{out}\left[t\right]$ represents the spike activity of *i*-th output neurons at *t*-th time point. *L*_*out*_ is the number of output neurons, equal to the number of classes. This definition gives a voting policy in that the neuron with the most spikes will be given the highest probability of determining the classification. Giving the one-hot teach signal $\vec{y}$ during training,


(43) $$\begin{array}{l} y_{i}=\{\begin{array}{cc} 1, & \;i\;\text{is target}\\ 0, & \;\text{otherwise} \end{array} \end{array}$$

The loss function *L* is then defined as the Cross-Entropy error, which will be minimized for all samples during training,


(44) $$\begin{array}{l} L=\overset{L_{out}}{\underset{i}{\sum }}y_{i}\log\left(p_{i}\right) \end{array}$$

## 3. Experiments and results

According to the mathematical assumptions and constraints, we first construct experiments for the discrete models at different discretization levels, and examine their stability issues under different timescales. Then, to evaluate the performance of the proposed SNN modules, we selected two neuromorphic datasets: N-MNIST (Orchard et al., 2015) and SHD (Cramer et al., 2020). They are used as experimental objects for classification error rates in neuromorphic tasks, including ablation experiments. In addition, we set up control experiments to analyze and discuss the significance of the three characteristics (MAP) to the model performance.

### 3.1. Timescale robustness analysis of discrete models

**Figure 7:**
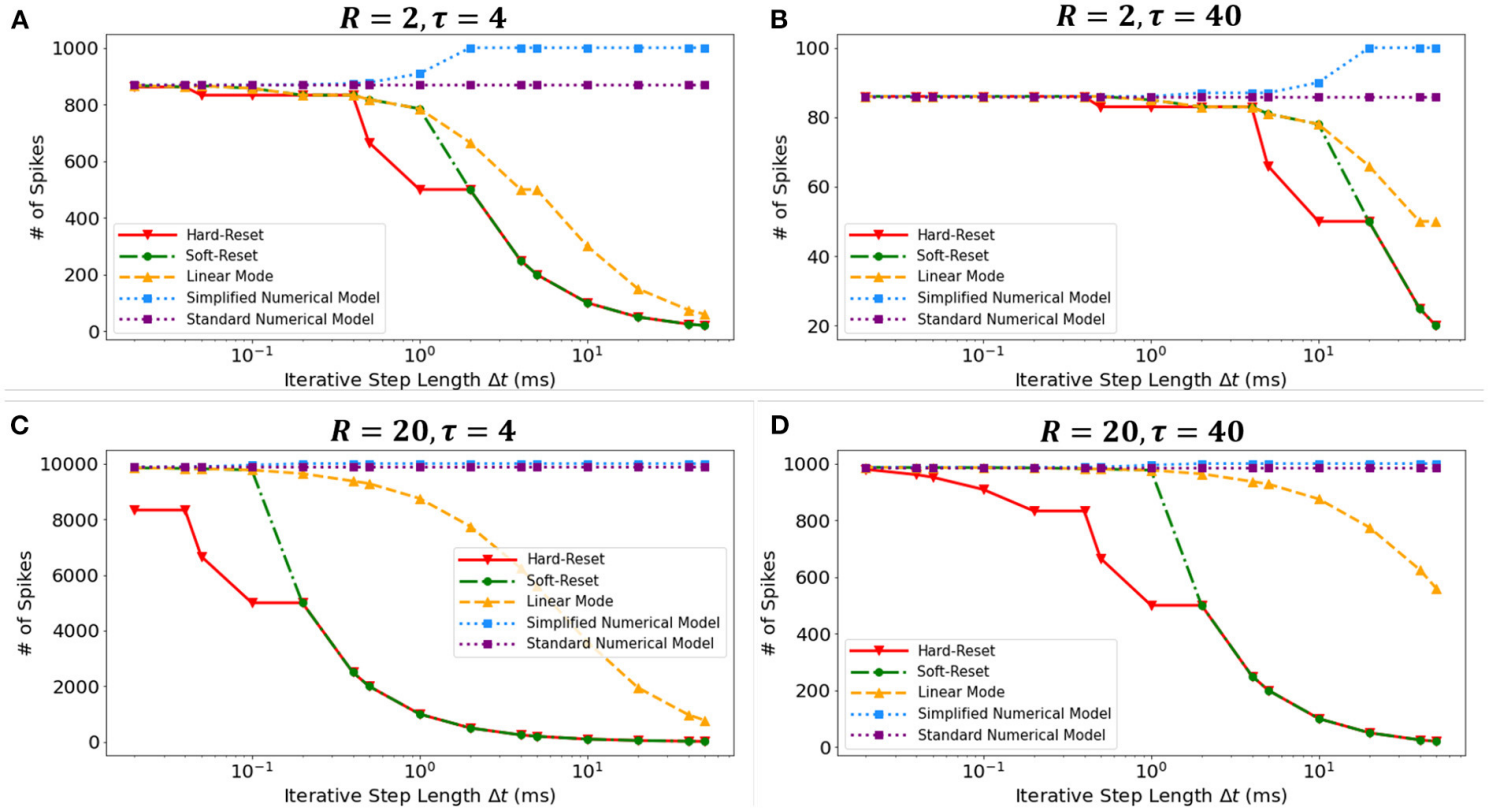
The numbers of spikes *via* Δ*t* with different discrete models. Four experiments with different τ and *R*: **(A)** *R* = 2Ω, τ = 4*ms*, **(B)** *R* = 2Ω, τ = 40*ms*, **(C)** *R* = 20Ω, τ = 4*ms*, **(D)** *R* = 20Ω, τ = 40*ms*.

We conducted a simulation experiment to validate the discretization-based theoretical analysis presented in Section 2.2. We set constant input current *I* = 1(*mA*), membrane potential threshold *V*_*th*_ = 1(*mV*), total runtime *T* = 1000(*ms*), membrane time constant τ ∈ {4(*ms*), 40(*ms*)}, and membrane resistance *R* ∈ {2Ω, 20Ω}. By simulating discrete models in Figure 3, we obtain the findings presented in the Figure 7. The results obtained under various τ and *R* confirm the derivation presented in Section 2.2.3. First, the Standard Numerical Model yields perfectly stable results, and the number of recorded spikes does not vary with Δ*t*. Comparing Figures 7A,B, the Simplified Numerical Model is a good approximation of the Standard Numerical Model when R is large; comparing Figures 7A,C, the Linear Mode of the MSP is closer to the Standard Numerical Model as τ increases. Notably, these findings indicate that Soft-Reset Model initially displays performance that is highly close to Linear Mode, but is restricted by binary transmission when Δ*t* is large, where the number of recorded spikes is inversely proportional to Δ*t*, indicating that the extension of the SSP to the MSP is rational.

### 3.2. Classification of neuromorphic datasets

To demonstrate the reliability of our approaches, we train our SNN models with spike-based datasets for image and sound classification and compare the achieved error rates with related works on SNN algorithms.

#### 3.2.1. Experiment setting and parameters initialization

The proposed model is built on the deep learning framework, PyTorch (Paszke et al., 2019), and the weights are initialed using the default Xavier Normal (Glorot and Bengio, 2010) method. Besides, we use Adam as the optimizer and Cross-Entropy as the criterion during training. Hyperparameters of experimental settings are shown in Table 1. We built fully connected networks for classification as the Multilayer Perceptron (MLP) structure.

**Table 1:** Hyperparameters setting.

**Network parameters**	**Description**	**Value**
*T*	# of Timesteps (N-MNIST/SHD)	15,50
*dt*	Timestep length (N-MNIST/SHD)	20, 16 ms
*r*	Learning rate (Adam)	10^−3^
*V*_*th*_	Threshold	2.0
*q*	Inhibition coefficient	1.2
τ_*decay*_	Initial decay factor	0.2
*delay*	Initial kernel delay factor	0.8
*a, b*	Initial kernel shape factors	*randam*[0.5, 1.0], *randam*[0.5, 1.0]
*kernel*_*size*	Kernel size of synaptic convolution	7

#### 3.2.2. Neuromorphic image dataset

N-MNIST (Orchard et al., 2015) is a neuromorphic version of MNIST digits, which contains 60,000 train samples and 10,000 test samples aligning with MNIST. The samples of N-MNIST are event-based spike signals captured by recording MNIST images displayed on an LCD screen using Dynamic Vision Sensors (DVS) mounted on a moving platform. The N-MNIST images record overall 300ms frames and have two channels that separately record brighter and darker brightness changes. We process the two channels in parallel with two groups of 400 hidden neurons, where the network architecture is (2 × 1156)−(400+400)−10.

#### 3.2.3. Neuromorphic sound dataset

Spiking Heidelberg Digits (SHD) (Cramer et al., 2020) is a spike-based speech dataset consisting of 0–9 spoken digits recordings in both English and German. The audio recordings are converted into spikes using an artificial inner ear model, transforming into temporal structures with 700 input channels. There are 8156 train samples and 2,264 test samples in SHD, and each of them lasts for at most 800 ms. SHD requires multiple layers to fit. Therefore, we built the architecture of 700−400−400−20 with two hidden layers of 400 neurons.

#### 3.2.4. Error rate comparison

We compare the obtained model performance with state-of-the-art SNN models, The results of N-MNIST are in Table 2 and SHD is in Table 3, including ablation experiments with MSP and SFSRM alone. The experimental results show that MAP-SNN can decrease the error rate by 0.16% on N-MNIST and 1.30% on SHD, which has achieved the highest performance among SNN-based algorithms under the same MLP structure. Furthermore, we observe that MSP and SFSRM can independently improve the model accuracy and be combined for significantly better performance, which supports the complementarity of MAP properties.

**Table 2:** Performance of different algorithms on N-MNIST.

**Model**	**Size of hidden layer**	**Error rate (%)**
Spiking-MLP (Cohen et al., 2016)	10,000	8.13
Spiking-CNN (Neil and Liu, 2016)	-	4.28
LSTM (Neil et al., 2016)	-	2.95
Phased-LSTM (Neil et al., 2016)	-	2.62
MLP (Lee et al., 2016)	800	2.20
Spiking-MLP (Lee et al., 2016)	800	1.26
STBP (Wu et al., 2018)	800	1.22
Spiking-MLP (Fang et al., 2021)	500-500	1.60
**This work (SSP)**	800	**1.60**
**This work (SSP+SFSRM)**	800	**1.43**
**This work (MSP)**	800	**1.11**
**MAP-SNN (MSP+SFSRM)**	800	**1.06**

The bold contents are the results of this work.

**Table 3:** Performance of different algorithms on SHD.

**Model**	**Size of hidden layer**	**Error rate (%)**
Spiking-MLP (Cramer et al., 2020)	–	52.5
SNN-base (Cramer et al., 2020)	–	28.6
R-SNN (Cramer et al., 2020)	–	16.8
R-SNN (Zenke and Vogels, 2021)	–	18.0
SNN-SoTa (Perez-Nieves et al., 2021)	–	17.3
SRNN (Yin et al., 2020)	–	15.6
Spiking-MLP (Fang et al., 2021)	400-400	14.3
**This work (SSP)**	400-400	**36.1**
**This work (SSP+SFSRM)**	400-400	**33.0**
**This work (MSP)**	400-400	**17.1**
**MAP-SNN (MSP+SFSRM)**	400-400	**13.0**

The bold contents are the results of this work.

#### 3.2.5. Visualization results

**Figure 8:**
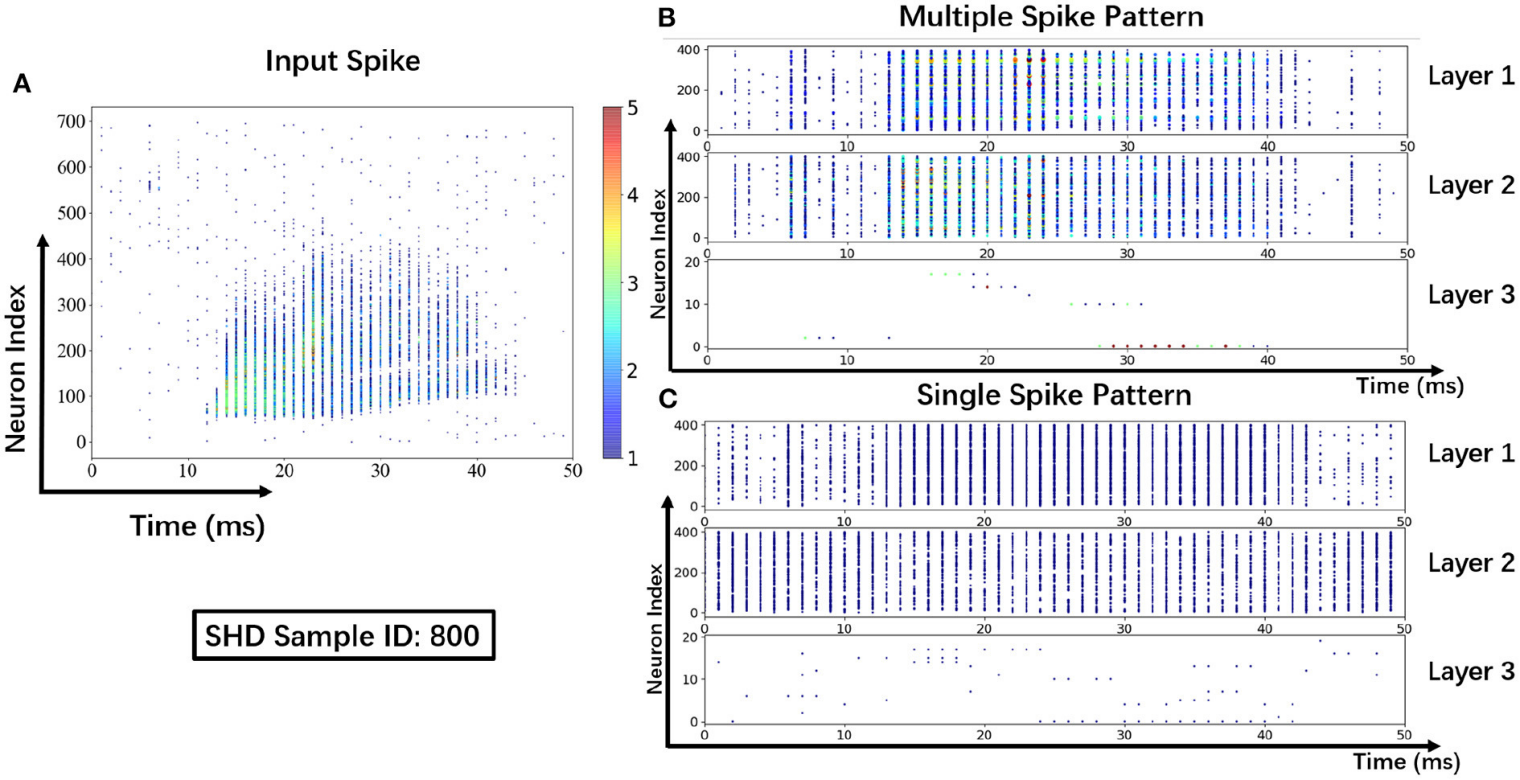
Spike raster plot: Visualization of spike transmission on SHD sample. **(A)** Input spikes. **(B)** Spike transmission in multiple spike pattern. **(C)** Spike transmission in single spike pattern.

To help understand the signal transmissions of SNNs and highlight the difference between the MSP and the SSP, we derive the spike raster plot of an SHD sample. The network structure consists of two hidden layers of 700−400−400−20. The plot shows the neural spike activities in layers on the 800-th SHD training sample with label 0 in Figure 8. The horizontal and vertical axes indicate the time interval and the indices of spiking neurons in layers. In detail, we implement the SSP based on STBP (Wu et al., 2018) for comparison with the proposed MSP. In Figure 8B, it is observed under MSP that spike activities are more concentrated in the temporal dimension, while there are fewer nearby sparse spikes in deep layers (Layer 2 and Layer 3). In contrast, Figure 8C shows that the spike channels of Layer 2 are almost full, which is because the neurons under SSP are “encouraged” to keep fire to maintain signal integrity within restricted binary signals.

### 3.3. Control experiments and performance analysis

To explore the potentials of the proposed MSP, SFA, and SFSRM, we carry out control experiments on N-MNIST and SHD datasets and discuss the impacts of MAP properties on improving model performance.

#### 3.3.1. The impact of multiplicity on discrete iteration

**Figure 9:**
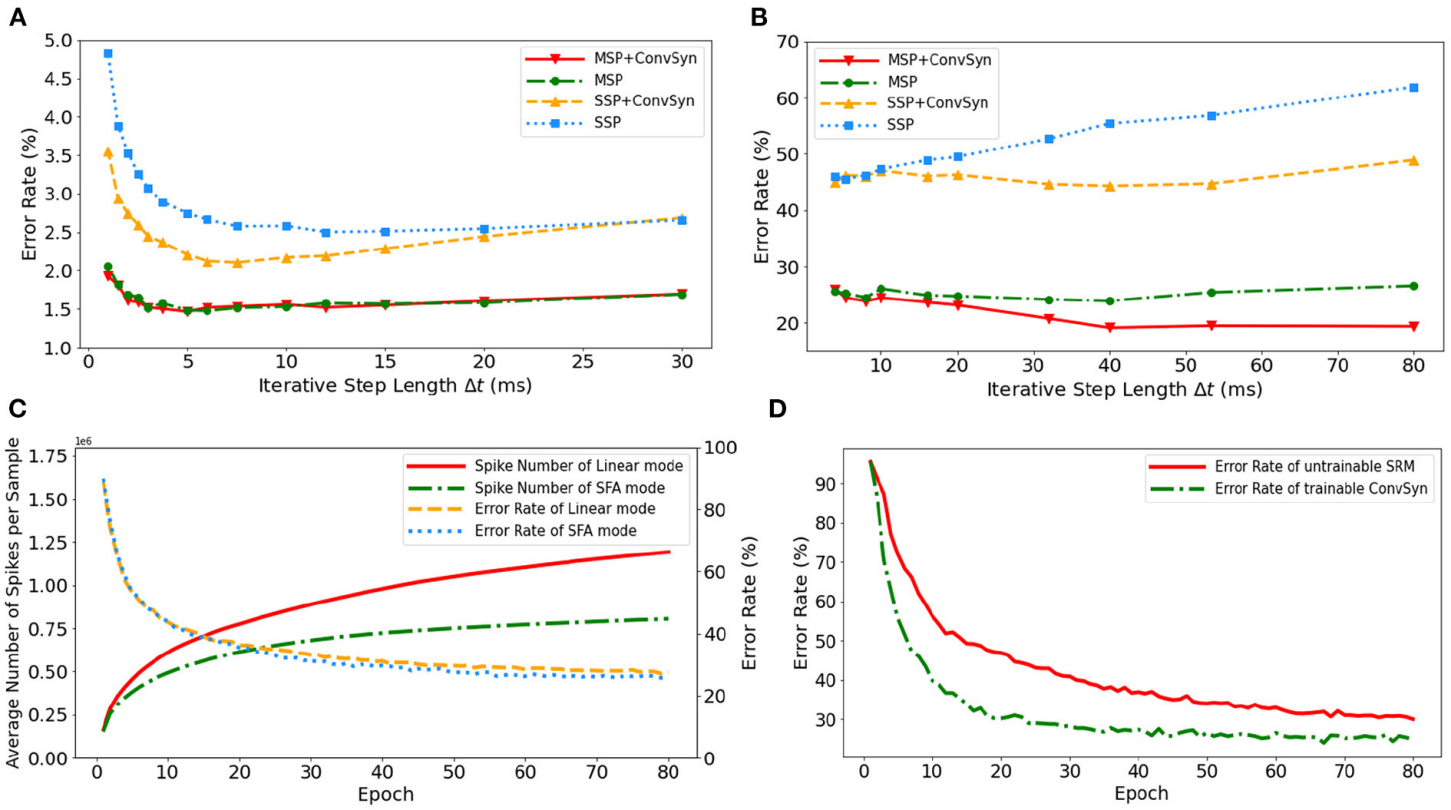
Experimental results. **(A)** Error rate curves among different iterative step lengths on N-MNIST. **(B)** Error rate curves among different iterative step lengths on SHD. **(C)** Control experiment of spike frequencies between SFA and Linear modes on SHD. **(D)** Control experiment of synaptic plasticity for performance improvement on SHD.

The selection of minimal iterative step lengths Δ*t* influences model performance in the discrete iterative models. For the sake of completeness of the analysis, we analyze this instability in the ablation experiments by building control experiments under MLP architecture with different iterative step lengths, as shown in Figures 9A,B. The experiments are based on N-MNIST and SHD, respectively, where the unified network structure is (2 × 1156)-200-10 on N-MNIST and 700-400-20 on SHD. With the MAP properties, the error rates of the model have been significantly improved. Compared with benchmark SSP, our MAP-SNN with the combination of MSP and SFSRM reduces error rates among different iterative step lengths by the range of (1.0, 2.8%) on N-MNIST, and (19, 41%) on SHD, which demonstrates the reliability of the proposed methods. Furthermore, the model trained with MSP keeps almost constant error rates across different Δ*t*, supporting that multiplicity alleviates the discretization problem and improves the model stability over time-iteration with different iterative step lengths.

#### 3.3.2. The impact of adaptability on spike efficiency

To demonstrate the effectiveness of SFA in spike reduction, we establish a set of controlled experiments on the SHD dataset with the 700-400-20 MLP structure. Figure 9C shows the error rates and spike numbers in the training process of models in both SFA mode and Linear mode. The experimental results show that SFA effectively suppresses spike activities by 1.48 × times while slightly improving model accuracy by 1.52%. In this case, the reduced signal transmissions helpfully decrease the amount of computation in synapses, which will help save the power consumption of neuromorphic hardware based on spike transmissions.

#### 3.3.3. The impact of plasticity on feature extraction

To highlight the importance of plasticity for feature extraction, we set up a control experiment to compare the SRM with and without the plasticity, as shown in Figure 9D. The untrainable SRM refers to the fixed SRM (the parameters fixed after initialization) and the trainable SFSRM refers to our proposed method in Section 2.4. The experiment is set on the SHD with the 700-400-20 MLP structure, where the SRM or SFSRM is inserted on 400 hidden neurons. Figure 9D shows the changes in model error rate and loss during the training epoch. The experimental results show that the plasticity allows the model to converge faster by 4.2% and reduces the error rate by 15.6% during epoch [10, 80]. This demonstrates the advantage of SFSRM in temporal feature extraction. We conclude that plasticity helps shorten the training process of models and improve the model's performance.

## 4. Discussion

In this work, we refer to the Multiplicity, Adaptability, and Plasticity (MAP) properties and model spike activities with Multiple-Spike Pattern (MSP), Spike Frequency Adaption (SFA), and State-Free Synaptic Response Model (SFSRM) that improve BP-based methods with better performance. For the spiking neural models, the existing methods rely on the neurons with the single-spike pattern (SSP) that only outputs the binary events (Lee et al., 2016; Wu et al., 2018; Cheng et al., 2020), introducing discrepancies between the discrete iterative simulation and biological network. To mitigate this discrepancy due to time discretization, we propose an MSP spiking neural model that supports the neurons to output the number of spike events. We set up the control experiments on neuromorphic datasets and tested SSP and MSP in the same MLP architecture. The results demonstrate that the SSP is sensitive to the selection of iterative time step Δ*t*, while the MSP is more robust under different time steps Δ*t* compared with the SSP, as shown in Figures 9A,B. Considering the smaller number of simulation timesteps and lower inference latency, the spiking neurons with multiple thresholds are proposed in recent works (Chowdhury et al., 2021; Xu et al., 2021), which refer to the linear mode of MSP implementation. The spike activity increases linearly as the increase of input currents in such linear mode MSP. More spike activities require more energy for spike transmission and subsequent operations. While a biological neuron fires with a reduced frequency over time under a constant stimuli (Benda and Herz, 2003). Therefore, the SFA mode was implemented under the MSP to reduce spike activities while keeping on the model accuracy shown in Figure 9C. Some published works have applied the SRM model in SNNs to capture the spike temporal information and thus achieved high performance (Jin et al., 2018; Shrestha and Orchard, 2018). However, they restrict the shape parameters inside the kernel function and need to expand the kernel among temporal domains to do the calculation step by step. In order to enrich the model representation power and make the SRM more compatible in deep frameworks, we propose to substitute the iterative calculation with convolution operations and allow all parameters inside the kernel to be learned for plastic synapses.

The implementability of the proposed MAP-SNN is a major concern. State-of-the-art neuromorphic chips, such as Tianjic (Pei et al., 2019), Darwin (Ma et al., 2017), and Loihi (Davies et al., 2018), support the single-spike pattern directly. The MAP-SNN model with a generalized definition is feasible to perform the on-chip inference on Tianjic Chip with proper hardware configuration. In detail, the neural models of multiple-spike patterns with SFA mode can be simplified at the hardware level by pre-setting a lookup table to determine the number of spike activities based on the value of the current membrane potential. Besides, in the axonal process of spike traveling, the Tianjic chip with ANN-SNN hybrid design can compatibly perform the integer transmission, supportive of the multiple-spike pattern. Further, at the axonal terminal, the state-free synapses are implemented by the low-pass filters, which transform the spike activities into synaptic currents. We believe that algorithms and hardware can be developed in tandem. The improvements in algorithms may also inspire the hardware design. The feasibility of MAP-SNN on Tianjic provides a potential development perspective for both spike-based algorithms and neuromorphic hardware.

The aforementioned discrete models (Figure 3) have great numerical calculation accuracy in simulation and inference, which may be used in the ANN-SNN conversion approach to narrow the gap between analytical and numerical solutions, as well as reduce network latency through time compression; this is an area worthy of further exploration in the future.

In conclusion, this work demonstrates the potency of effectively modeling spike activities, revealing a unique perspective for researchers to re-examine the significance of biological facts.

## Data availability statement

Publicly available datasets were analyzed in this study. This data can be found here: the datasets SHD/N-MNIST for this study can be found in the https://zenkelab.org/resources/spiking-heidelberg-datasets-shd/ (Spiking Heidelberg Datasets), and https://www.garrickorchard.com/datasets/n-mnist (Neuromorphic-MNIST).

## Code availability statement

The source code of MAP-SNN can be found in the Github repository https://github.com/Tab-ct/MAP-SNN.

## Author contributions

CY proposed the idea. CY and YD designed and did the experiments. CY, MC, and AW wrote the manuscript, then GW, AW, and EL revised it. AW directed the projects and provided overall guidance. All authors contributed to the article and approved the submitted version.

## Funding

This work was supported in part by the Fundamental Research Funds for the Central Universities under Grant 2-2050205-21-688 and in part by the Zhejiang Provincial Natural Science Foundation Exploration Youth Program under Grant LQ22F010011.

## Conflict of interest

The authors declare that the research was conducted in the absence of any commercial or financial relationships that could be construed as a potential conflict of interest.

## Publisher's note

All claims expressed in this article are solely those of the authors and do not necessarily represent those of their affiliated organizations, or those of the publisher, the editors and the reviewers. Any product that may be evaluated in this article, or claim that may be made by its manufacturer, is not guaranteed or endorsed by the publisher.
